# Radiation Changes the Metabolic Profiling of Melanoma Cell Line B16

**DOI:** 10.1371/journal.pone.0162917

**Published:** 2016-09-15

**Authors:** Lige Wu, Zixi Hu, Yingying Huang, Yating Yu, Wei Liang, Qinghui Zheng, Xianing Huang, Yong Huang, Xiaoling Lu, Yongxiang Zhao

**Affiliations:** 1 National Center for International Research of Biological Targeting Diagnosis and Therapy, Guangxi Key Laboratory of Biological Targeting Diagnosis and Therapy Research, Collaborative Innovation Center for Targeting Tumor Diagnosis and Therapy, Guangxi Medical University, Nanning, Guangxi 530021, China; 2 The Department of Immunology, Guangxi Medical University, Nanning, Guangxi 530021, China; Istituto Superiore di Sanità, ITALY

## Abstract

Radiation therapy can be an effective way to kill cancer cells using ionizing radiation, but some tumors are resistant to radiation therapy and the underlying mechanism still remains elusive. It is therefore necessary to establish an appropriate working model to study and monitor radiation-mediated cancer therapy. In response to cellular stress, the metabolome is the integrated profiling of changes in all metabolites in cells, which can be used to investigate radiation tolerance mechanisms and identify targets for cancer radiation sensibilization. In this study, using ^1^H nuclear magnetic resonance for untargeted metabolic profiling in radiation-tolerant mouse melanoma cell line B16, we comprehensively investigated changes in metabolites and metabolic network in B16 cells in response to radiation. Principal component analysis and partial least squares discriminant analysis indicated the difference in cellular metabolites between the untreated cells and X-ray radiated cells. In radiated cells, the content of alanine, glutamate, glycine and choline was increased, while the content of leucine, lactate, creatine and creatine phosphate was decreased. Enrichment analysis of metabolic pathway showed that the changes in metabolites were related to multiple metabolic pathways including the metabolism of glycine, arginine, taurine, glycolysis, and gluconeogenesis. Taken together, with cellular metabolome study followed by bioinformatic analysis to profile specific metabolic pathways in response to radiation, we deepened our understanding of radiation-resistant mechanisms and radiation sensibilization in cancer, which may further provide a theoretical and practical basis for personalized cancer therapy.

## Introduction

Ionizing radiation is the main cause of death for cancer cells in radiation therapy, but many cancers for example melanoma are not sensitive to radiation therapy, resulting in poor clinic effects [1, 2]. Radiotherapy is one of the most important methods of cancer treatment along with surgery and chemotherapy. Radiotherapy was administered to shrink tumor in advanced melanoma, or prevent tumor relapse after surgical treatment [3, 4].

In addition to killing cancer cells, radiation therapy also leads to damage of normal cells and tissues. It is therefore an urgent medical concern to protect the normal cells in addition to killing cancer cells as many as possible. To this end, studies on radiosensitization are receiving more and more attention in radiobiology [5, 6]. As a comprehensively used reagent, a radiosensitizer can facilitate the sensitivity of cancer cells in response to radiation, which accordingly promotes the effects of therapy by increasing radiation-mediated cancer cell death [7, 8]. Initially, radiosensitizers were used in radiation-resistant anaerobic cells in solid tumors, but this application has now extended to other cell types in cancers [9]. Though a radiosensitizer is effective in increasing the effectiveness of radiation therapy for cancer, most radiosensitizers are chemotherapy drugs, with unavoidable toxicity for normal cells. Therefore, the search for low-toxic and highly effective radiosensitization is an urgent need in tumor radiation therapy.

The nuclear magnetic resonance (NMR) spectrum–based cellular metabolome analysis is a novel method to assist radiosensitization study in tumor radiation therapy. ^1^H NMR analysis is an ideal high throughput method to detect small-metabolites in biological samples [10, 11]. With pattern recognition, ^1^H-NMR-based metabolome analysis screens differentially expressed metabolites between different samples [12]. Followed by bioinformatics analysis on the basis of a public database, the changed metabolic pathways can be further identified.

In this study, using radiation-tolerant mouse melanoma cell line B16 as research model [13], cells were first radiated with sublethal doses of X-ray radiation; then cellular metabolites were collected, which were further analyzed by 600 MHz ^1^H NMR to screen differentially expressed metabolites. Following bioinformatic analysis for those quantified metabolites, potential targets of disturbed metabolic pathway can be used for radiosensitization study to discover high efficacy drugs for sensitization in radiotherapy. The underlying mechanism of metabolome changes and radiation resistance in B16 cells was discussed.

## Experimental Procedures

### Cell line and cell culture

Mouse melanoma cell line B16 (obtained from China Center for Type Culture Collection, Wuhan University, Wuhan, China) was maintained in a complete RPMI-1640 medium supplemented with 10% fetal bovine serum and 1% Penicillin-Streptomycin. After a sublethal dose of X-ray radiation (18 Gy) using RS2000 X-ray irradiator (Rad Source Technologies Inc., GA, U.S.), 1×10^6^ cells were subcultured in 25 cm^2^ plates in 5% CO_2_ at 37°C. Untreated cells cultured in the same conditions were used as control cells. Cells were harvested after 48 h culture for further experiments. The experiment was repeated 3 times.

### Cellular metabolites collection

One-step quenching and extraction were applied as previously described to cell samples [14, 15]. Briefly, after the removal of cell culture medium, the cells were washed twice with ice-cold PBS, and then the pellets were quenched by 2 mL −20°C methanol (HPLC grade, Kermel Chemical Reagent, Tianjin, China). Afterwards, the cells were scraped and transferred into a 15-mL centrifugation tube, followed by adding 2 mL −20°C chloroform and ice-cold ddH_2_O (V/V/V 1:1:0.7). Cell lysates were mixed by vortex for 5mins and left stand for 15 mins, and then centrifuged with 14,000 g for 30 min at 4°C, generating two-phase extraction. The aqueous phase was lyophilized and then dissolved into 450 μL D_2_O with 50 μL buffer (1.5 M K_2_HPO_4_, 0.375 M NaH_2_PO_4_, 0.1% TSP, 0.2% NaN_3_, pH 7.4) [16]. After being mixed with vortex, undissolved substances were removed by 14,000 × g centrifugation for 5 mins at 4°C and the supernatant was transferred into a 5-mm nuclear magnetic tube for NMR analysis.

### 1H Nuclear magnetic spectra collection

^1^H NMR analysis was performed by a Bruker AVANCE III 600 MHz NMR spectrometer at 298.15K. The samples were analyzed by pre-saturated pressed water peak pulse sequence noesypr1d ([RD-90°-t1-90°-tM-90°-ACQ]). Relaxation delay was set as 3 s, t1 4 μs, t_M_ 120 ms, sampling time 1.64 s. Two hundred fifty-six free induction decays (FIDs) were collected by TOPSPIN software. The data point at 32 K was kept and adjusted to 64 K with spectral width 10 kHz. Spectral data were acquired from Fourier transform, by a window from all FIDs multiplying exponential function of line width 1 Hz.

### Spectral data preprocessing

Phase and baseline of all NMR spectra were adjusted by MestReNova software, and the TMSP (Trimethylsilylpropanoic acid) peak was set as 0 ppm. The text was derived after calculating piecewise integration, with 0.002 ppm as the interval. Data preprocessing was performed with self-prepared MATLAB script. Data points within 0.6–9.5 ppm were kept and the peak water interval (4.5–5.2 ppm) was removed. For each spectrum, 4450 bins were kept. To reduce the difference due to different sample concentration, the dilution factor of each sample was calculated according to a control spectrum after probabilistic quotient normalization (PQN) [17]

Pseudo two-dimensional spectra were drawn, based on preprocessing the data of statistical total correlation spectroscopy (STOCSY), which indicated correlation factors among each chemical shift [18]. To reduce the ratio of false positives, the threshold of the correlation factor r was calculated by an adjusted p value and number of varieties by Bonferroni.

### Dimensionality reduction and pattern recognition analysis

Dimensionality reduction and pattern recognition analysis was carried out by imputing preprocessed data into the software SIMCA-P+ (Ver. 12.0, Umetrics, Umeå, Sweden). To reduce the difference due to different sample concentrations, data were converted by Pareto [19].

Firstly, principal component analysis (PCA) was applied to perform data-dimensionality reduction and get a data preview [20]. Afterwards, we use partial least squares discriminant analysis (PLS-DA), a supervised pattern recognition method, to determine the modeling that generated the greatest difference between radiated sample and untreated sample. Grouping information used as response variable Y in calculating PLS-DA model. Q^2^ and R^2^were generated by software calculation to evaluate the degree of fit and forecasting ability of model. To avoid over-fitting, 7-fold cross-validation was performed for 400 repeats [21–23].

The validated model was formally established by orthogonal projections to latent structures discriminant analysis (OPLS-DA), significant components were separated into one predictive component, t1, to describe the differences and one or more orthogonal components to filtering the irrelevant noise, the two datasets may be the most distinguished on score chart [24]. After exporting the model correlation factor p(corr) and variable importance projection (VIP) as well as the load value, the loading diagram was drawn after backtracking transformation, and then differential metabolites were identified.

### Univariate analysis

Univariate analysis was performed on those differential metabolites identified by pattern recognition and substances identified by STOCSY. The relative concentration of differential metabolites was acquired by calculating the integration of spectra peaks. According to the p value calculated by the Mann–Whitney test in GraphPad Prism 6 (Ver. 6.01, GraphPad Software, Inc., CA, U.S.), the statistical significance of the differential metabolite was further verified.

### Pathway enrichment analysis

The names of the metabolites and their KEGG IDs were matched using MetaboAnalyst (http://www.metaboanalyst.ca/) [25]. Pathway enrichment analysis was performed using MBRole [26]. The relevant pathways were acquired by inputting metabolite KEGG ID and running a Hypergeometric Test. The metabolic pathway illustration was generated by Cytoscape and MetScape [27].

## Results

### Radiation changed the metabolic profiling of B16 cells

**Fig 1:**
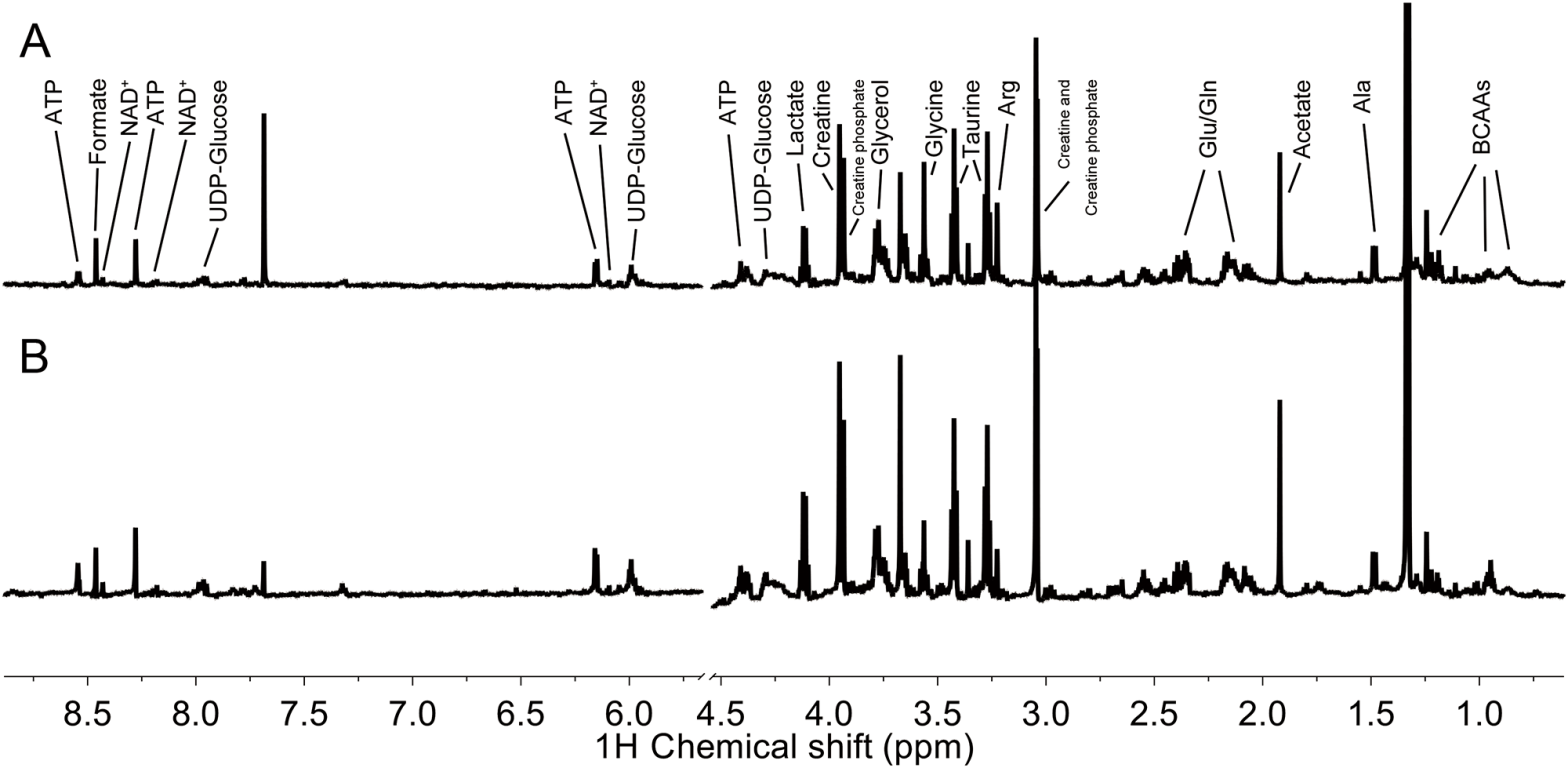
Representative spectra and peak assignment. **A:** NMR spectra of irradiated B16 cells; **B:** NMR spectra of untreated B16 cells. Spectrum peak with chemical shift δ 0.5–8.6 ppm was kept and water peak as removed. The main metabolites indicated in the spectra were identified by Chenomx NMR Suite software and statistical total correlation spectroscopy (STOCSY).

To identify the representative metabolites in B16 cells in response to radiation, we firstly compared ^1^H NMR spectra from X-ray radiated B16 cells and untreated control cells. The representative spectrum of cellular soluble metabolite from X-ray radiated B16 cells and untreated control cells was indicated in Fig 1A and 1B, where the spectrum peak with δ0.5–8.6 ppm chemical shift was kept and water peak was removed. With ChenomxNMR Suite software analysis and statistical total correlation spectroscopy (STOCSY), major metabolites in the spectrum were confirmed. Chemical shifts and corresponding groups were summarized in Table 1.

**Table 1:** Peak assignments and integral regions.

Metabolite	Assignment	δ (ppm)		Untreated		Irradiated	
		From	To	Mean	SD	Mean	SD
Acetate	α-CH_3_ (d)	1.93	1.91	448.79	50.31	341.49	61.04
Ala	β-CH_3_ (d)	1.50	1.47	155.38	27.86	177.45	7.02
Asp	α-CH (dd)	2.69	2.64	146.49	17.89	141.64	20.93
	β-CH_2_ (dd)	2.71	2.69	40.42	12.33	37.25	10.26
	β-CH_2_ (dd)	2.84	2.79	49.80	7.61	47.41	3.81
ATP	5’-CH_2_ (m)	4.44	4.40	284.96	30.21	207.76	16.93
	1’-CH (d)	6.17	6.14	205.79	9.59	170.23	18.21
	2-CH (s)	8.29	8.27	143.62	14.75	117.74	9.66
Choline	N-CH_3_ (s)	3.23	3.22	100.98	9.95	203.64	9.29
Creatine	N-CH_3_ (s)	3.04	3.03	503.70	37.06	435.52	58.59
	α-CH_2_ (s)	3.94	3.92	387.04	17.13	312.79	25.32
CP	N-CH_3_ (s)	3.06	3.04	927.33	103.48	740.98	68.27
	α-CH_2_ (s)	3.97	3.94	624.26	33.22	509.38	41.90
Formate	H (s)	8.47	8.45	113.10	17.25	96.75	16.06
Glu	β-CH_2_ (m)	2.10	2.02	329.62	43.42	327.81	37.01
	γ-CH_2_ (m)	2.38	2.32	402.98	14.69	429.76	22.17
Glucose	1-CH (d)	5.25	5.23	28.29	9.19	11.23	9.49
Glutamine	γ-CH_2_ (m)	2.48	2.43	150.35	21.21	148.91	20.88
Glutathione	Glu-γ-CH_2_ (m)	2.59	2.54	220.70	33.58	214.76	28.23
	Cys-β-CH_2_ (m)	3.01	2.95	104.20	22.41	115.49	26.55
Glycerol	CH_2_ (m)	3.56	3.54	121.56	19.39	139.28	29.26
	CH_2_ (m)	3.59	3.57	124.95	5.70	144.05	24.38
	CH_2_ (m)	3.67	3.64	337.51	44.32	394.20	32.42
Glycine	α-CH_2_ (s)	3.57	3.56	135.84	3.69	240.80	11.82
Ile	β-CH_3_ (d)	1.03	1.00	43.81	8.75	36.68	13.68
Lactate	CH_3_ (d)	1.35	1.31	2737.81	98.25	1950.38	238.82
	CH (q)	4.14	4.09	527.75	36.47	390.44	49.93
Leu	γ-CH_3_ (dd)	0.98	0.95	176.43	9.18	105.66	9.56
	CH_2_ and CH (m)	1.77	1.71	149.40	20.52	54.86	27.83
Methanol	CH_3_ (s)	3.37	3.35	262.02	145.77	92.50	28.61
Pyruvate	CH_3_ (s)	2.40	2.39	95.97	14.72	84.79	14.17
Succinate	2 × CH_2_ (s)	2.41	2.40	43.69	10.14	40.02	5.73
Taurine	N-CH_2_ (t)	3.29	3.25	844.63	35.21	851.09	33.52
	S-CH_2_ (t)	3.44	3.41	842.21	26.76	828.75	35.74
UDP-glucose	Glucose 4-CH (m)	3.50	3.45	127.82	33.61	73.28	19.22
	Glucose 6-CH_2_ (m)	3.90	3.88	46.02	13.02	42.22	9.58

(s) singlet, (d) doublet, (t) triplet, (q) quartet, (dd) doublet of doublet, (m) multiplet

**Fig 2:**
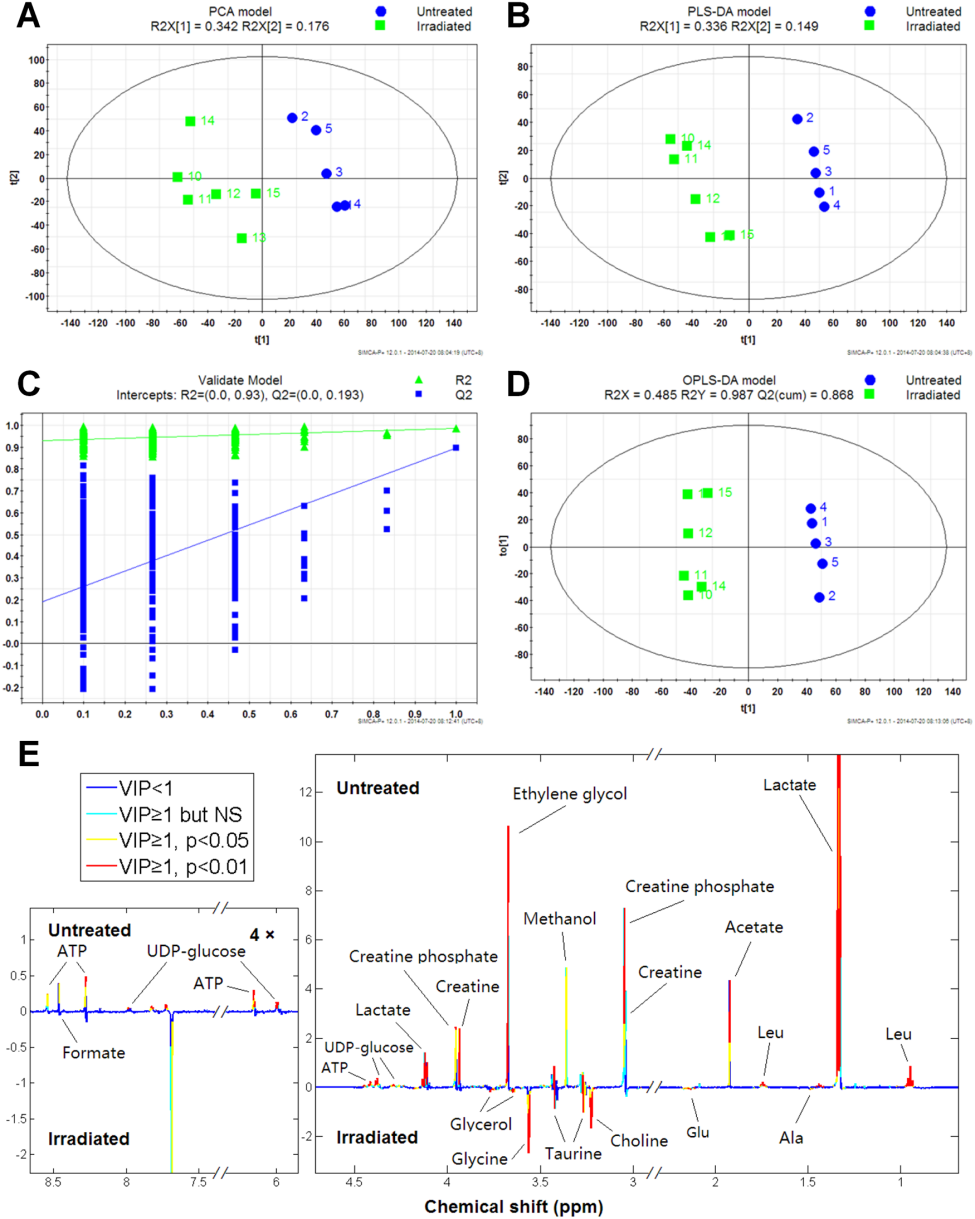
Multivariate analysis and back-scaled loading plot. Differential “metabogram” plotted according to correlation coefficient and VIP value. **A:** Radiated and untreated B16 cells were distinguished in principal component score chart, which indicated that the metabolic profiling of radiated B16 cells was different with that of untreated cells, t[1] and t[2] are scores on PC1 and PC2, respectively. **B:** Score chart of partial least squares discriminant analysis model distinguished radiated B16 cells and untreated B16 cells. **C:** 400 cross validations indicated little over-fitting and a reliable model. **D:**OPLS-DA model distinguished radiated and untreated B16 cells. **E:** Differential metabolites were distinguished in radiated and untreated cells according to VIP, relevant index r and loading value.

Principal component analysis (PCA) is an unsupervised pattern recognition algorithm, which is able to effectively eliminate the interference of artificial factors and indicates the sample distribution in principal component space. Taking advantage of this, all data were analyzed by PCA. A score plot of the first two principal components are shown in Fig 2A, radiated B16 cells and untreated B16 cells can be distinguished by first two principal components (PC1 and PC2), suggesting a metabolic profiling difference between radiated B16 cells and untreated B16 cells.

Next, a supervised pattern recognition algorithm was applied using PLS-DA. As shown in Fig 2B, radiated and untreated cells were able to be distinguished in the principal component in the score chart. After 400 cross-validations, little over-fitting was observed and the data presented good predictive feature in the model (Fig 2C). On the basis of a reliable PLS-DA model, orthogonal partial least squares discriminant analysis (OPLS-DA) model was established by rotated principal–component projection to filter irrelevant information, and the predication of principal components in the samples of two individual groups showed the greatest distinction (Fig 2D). Furthermore, differentially expressed metabolites between the two group samples were distinguished by analyzing the VIP of each variety, the correlation coefficient r, and the loading value. According to VIP and the correlation coefficient r, a metabogram was presented that clearly indicated changes in metabolites in different treatments. In summary, in response to radiation, the contents of leucine, lactate, acetate, creatine, creatine phosphate, methanol, ethylene glycol, UDP-glucose, ATP, and formate were decreased in cells, while the contents of alanine, glutamate, taurine, choline, glycerol, and glycine were increased in cells.

### Radiation induced the content change in metabolites in B16 cells

**Fig 3:**
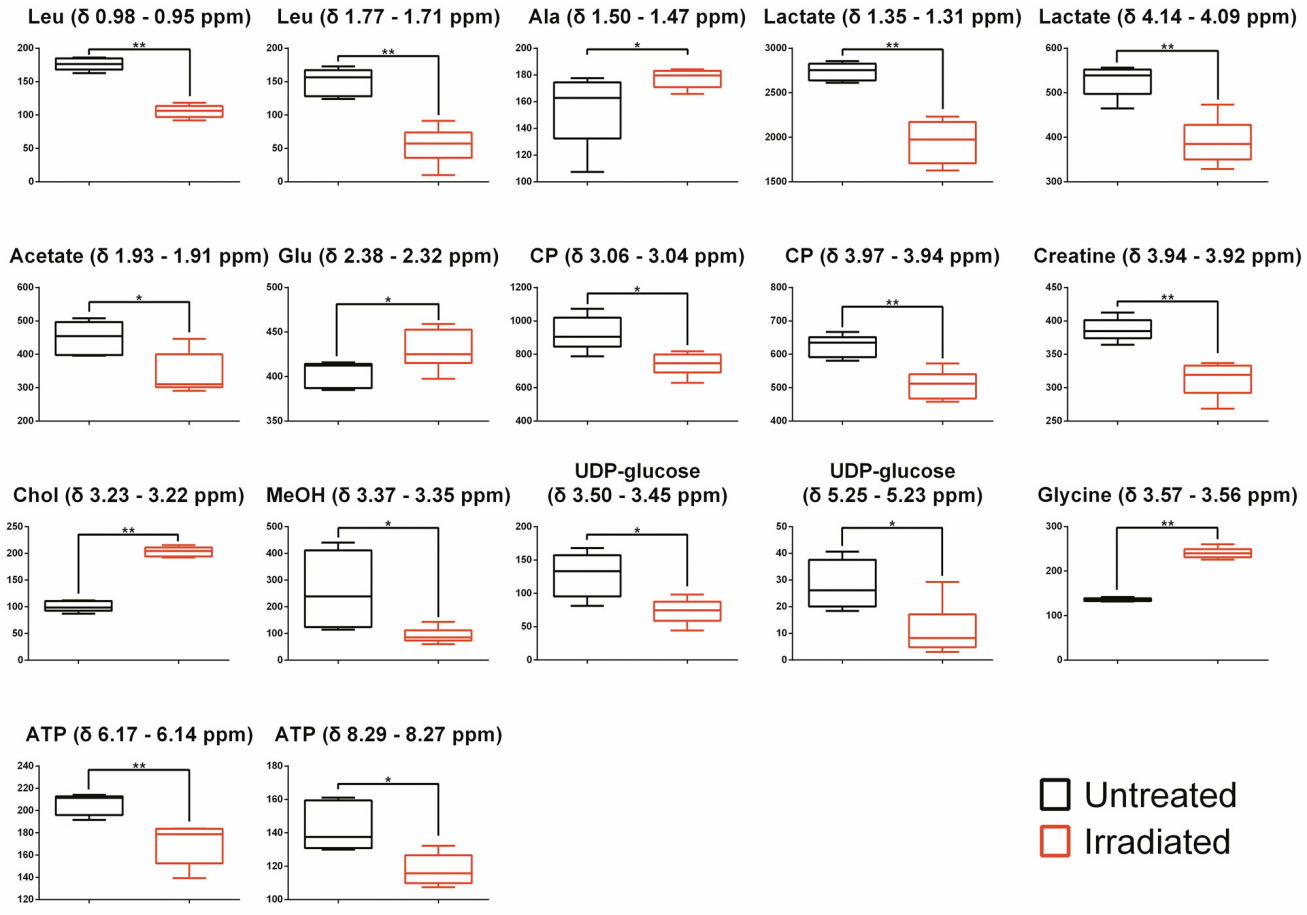
Relative concentration determined by the area under the peaks. Leu, Leucine; Ala, Alanine; CP, Creatine phosphate; Chol, Choline; MeOH, Methanol. Calculation ofp values were done by the Mann–Whitney test, * p < 0.05, ** p < 0.01.

By calculating the spectra peaks of corresponding metabolites, the relative concentration of metabolites was acquired. Single-variable analysis was performed to validate the statistical difference. For each metabolite, 1 or 2 non-overlapping or little-overlapping spectrum peaks were selected as specific spectrum peaks to calculate the integral area by which the relative concentration of each metabolite was acquired (Table 1). The relative concentration of differentially expressed metabolites was performed in a non-parametric statistical test of single variable to generate a p value by the Mann–Whitney test, and the differentially expressed metabolites with statistical significance were indicated in Fig 3 (* p < 0.05, ** p < 0.01). Consistent with the results of multivariable analysis, in response to radiation, the contents of alanine, glutamate, choline and glycine were increased, while the content of leucine, lactate, acetate, creatine, creatine phosphate, methanol, UDP-glucose, and ATP were decreased.

### Radiation changed the metabolic pathways and network in B16 cells

Through pattern recognition analysis and single variable analysis, in total 12 differentially expressed metabolites in B16 cells were identified upon radiation, including 4 content increased metabolites (alanine, glutamate, choline, and glycine) and 8 content-decreased metabolites (leucine, lactate, acetate, creatine, creatine phosphate, methanol, UDP-glucose and ATP). Correspondingly, for those content-changeable metabolites, their KEGG IDs and changes were listed in Table 2.

**Table 2:** Radiation related metabolites in B16 cells.

Match	KEGG	Change direction
L-Alanine	C00041	▲
L-Glutamate	C00025	▲
Choline	C00114	▲
Glycine	C00037	▲
L-Leucine	C00123	▼
L-Lactic acid	C00186	▼
Acetic acid	C00033	▼
Phosphocreatine	C02305	▼
Creatine	C00300	▼
Methanol	C00132	▼
Uridinediphosphate glucose	C00029	▼
Adenosine triphosphate	C00002	▼

**Fig 4:**
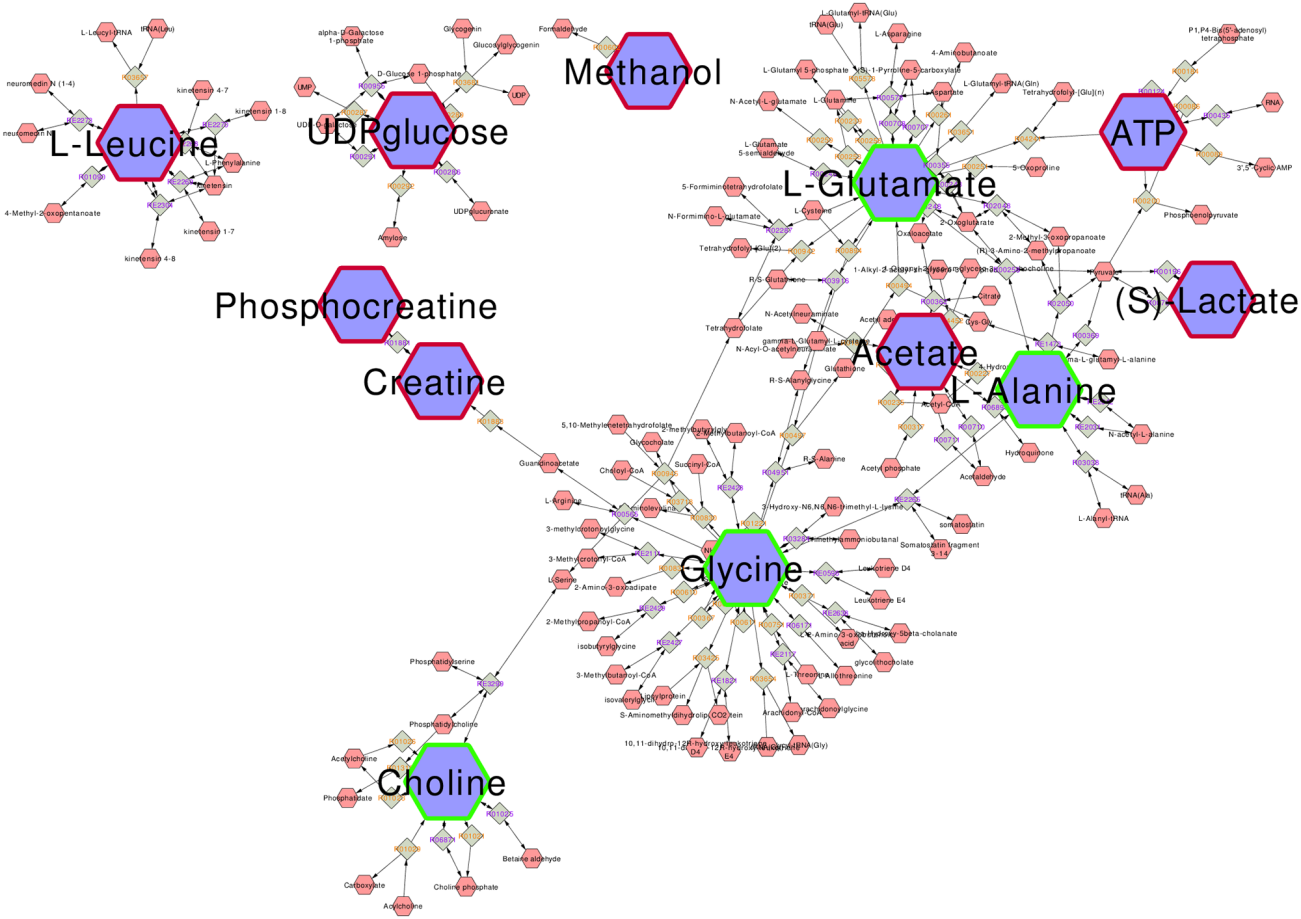
Radiation-associated metabolic network generated by MetScape. Red frames indicate increased metabolites in radiated B16 cells and green frames indicate decreased ones.

In MBRole, pathway enrichment analysis indicated that differentially expressed metabolites were involved in multiple metabolic pathways, including the metabolism of glycine, taurine, arginine and alanine (Table 3). Using MetScape, metabolic network illustration shows the connection of these metabolites (Fig 4), where hexagons represente metabolites, red frames indicate the decreased metabolites, green frames indicate increased metabolites, squares indicate KEGG ID, rounded rectangles represent enzymes, and blue represents regulative genes.

**Table 3:** Influenced pathways generated from MBRole by enrichment analysis (adjusted p values< 0.05).

id	label	pval	adj. pval	in bckgnd	in set	%
mmu02010	ABC transporters	6.16E-06	3.26E-04	90	5	41.7
mmu01100	Metabolic pathways	1.95E-05	5.17E-04	1455	12	100
mmu00970	Aminoacyl-tRNA biosynthesis	7.71E-05	1.36E-03	75	4	33.3
mmu00260	Glycine, serine and threonine metabolism	4.85E-04	6.42E-03	49	3	25
mmu00430	Taurine and hypotaurine metabolism	1.89E-03	1.93E-02	20	2	16.7
mmu00330	Arginine and proline metabolism	2.19E-03	1.93E-02	82	3	25
mmu00250	Alanine, aspartate and glutamate metabolism	2.72E-03	2.06E-02	24	2	16.7
mmu00910	Nitrogen metabolism	3.19E-03	2.12E-02	26	2	16.7
mmu00450	Selenoamino acid metabolism	4.24E-03	2.32E-02	30	2	16.7
mmu00010	Glycolysis / Gluconeogenesis	4.53E-03	2.32E-02	31	2	16.7
mmu00620	Pyruvate metabolism	4.82E-03	2.32E-02	32	2	16.7
mmu00680	Methane metabolism	5.43E-03	2.40E-02	34	2	16.7
mmu00480	Glutathione metabolism	6.76E-03	2.76E-02	38	2	16.7
mmu04080	Neuroactive ligand-receptor interaction	7.73E-03	2.93E-02	128	3	25
mmu01110	Biosynthesis of secondary metabolites	2.94E-02	1.04E-01	1023	7	58.3
mmu00230	Purine metabolism	3.64E-02	1.21E-01	92	2	16.7

**Fig 5:**
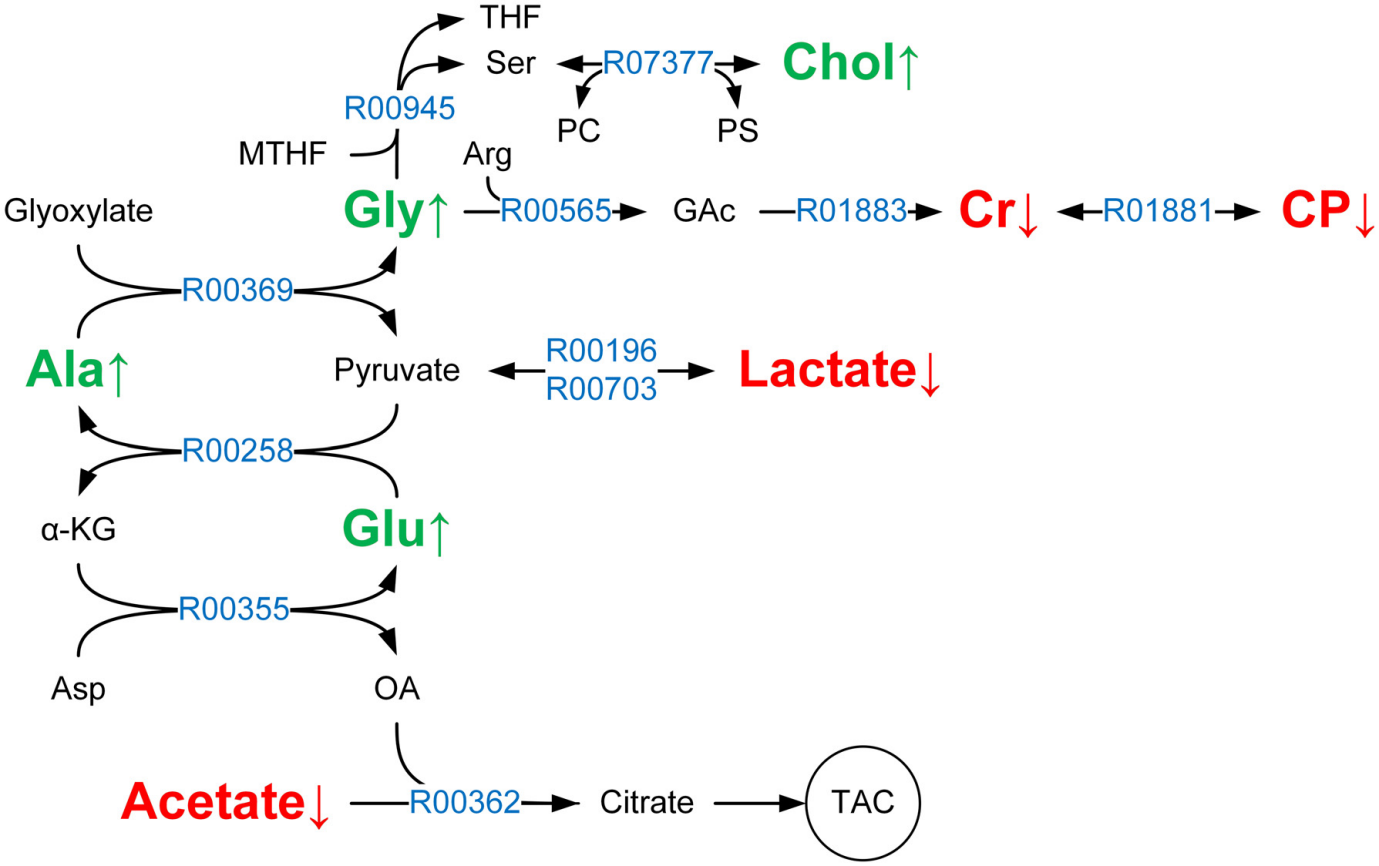
Altered pathways in radiated B16 cells. PC, Phosphatidylcholines; PS, Phosphatidylserine; MTHF, 5,10-methylenetetrahydrofolate; THF, Tetrahydrofolate; GAc, Guanidinoacetate; Arg, Arginine; α-KG, α-ketoglutarate, also known as oxoglutarate; Asp, aspartate; OA, Oxaloacetate; TAC, tricarboxylic acid cycle, also known as citrate cycle. Red represents increased metabolites and green represents decreased metabolites in response to radiation.

From metabolic pathway and network analysis, we observed that glutamate and pyruvate were converted into alanine and α-ketoglutarate by catalysis of alanine aminotransferase (ALT); Alanine and glyoxalic acid were converted into glycine and pyruvate by catalysis of Alanine-glyoxylate transaminase (AGT) (Fig 5). For those catalytic reactions, key enzymes and the EC number were listed in Table 4.

**Table 4:** Key enzymes in altered pathways.

Reaction	Enzyme	Abbr.	EC
R00703	L-lactate dehydrogenase	LDH	1.1.1.27
R00196	L-lactate dehydrogenase (cytochrome)		1.1.2.3
R01883	Guanidinoacetate N-methyltransferase	GAMT	2.1.1.2
R00945	Glycine hydroxymethyltransferase		2.1.2.1
R00565	Glycine amidinotransferase	AGAT	2.1.4.1
R00355	Aspartate transaminase	AST	2.6.1.1
R00258	Alanine transaminase	ALT	2.6.1.2
R00369	Alanine-glyoxylate transaminase	AGT	2.6.1.44
R01881	Creatine kinase	CK	2.7.3.2
R07377	Phosphatidylserine synthase 1		2.7.8.-
R00362	Citrate (pro-3S)-lyase		4.1.3.6

## Discussion

To investigate the impacts of radiation, the response of the metabolome in B16 cells to radiation were analyzed. Though metabolome analysis was performed in many clinic tissues or biofluids [28, 29], using cultured cell samples as a metabolic research model has multiple advantages, as not only can one exclude individual differences in clinic samples and tumor tissue heterogeneity, but also they are stable and have a high controllability; therefore, cell samples are appropriate for preliminary studies [30].

Radiation therapy can generate free radicals and reactive oxygen species (ROS) from X-ray radiation, which induces DNA damage in M-phase and G2-phase cells and subsequently blocks cells in the G2/M phase. By inducing cell cycle arrest–mediated apoptosis, cancer cells are killed with radiation therapy [31–33]. Nevertheless, it has been shown that multiple mechanisms lead to cancer tolerance of radiation therapy (e.g., anti-apoptosis [34], DNA damage repair [35], cell cycle regulation [36]). Previous studies indicated that radiation had little impact on the B16 cell cycle, and the observation of γH2AX foci showed that the ratio of DNA double strand break in radiated B16 cells was higher than that of untreated cells [37]. Impaired DNA double-strand break repair by chemical castration in prostate cancer had an improved response to radiotherapy [38]. In this study, the content of glycine in radiated B16 cells was increased. It is known that glycine can react with 5,10-methylenetetrahydrofolate by catalysis of glycine hydroxymethyltransferase to generate serine and tetrahydrofolic acid, and the latter is an important coenzyme of DNA synthesis process. This conclusion suggested that radiation tolerance in B16 cells may arise from DNA damage repair. Accordingly, by activating specific metabolic pathways to block DNA damage repair, radiation-tolerant cancer cells can be killed, which may possibly be an effective way of radiotherapy sensitization.

In response to radiation in B16 cells, the contents of glutamate, alanine, glycine, and choline were increased, which indicated that the activity of aspartate transaminase (AST), alanine transaminase (ALT), and alanine-glyoxylate transaminase (AGT) was elevated. Therefore, we speculate that, with a series of biochemical reactions upon the catalysis of those enzymes, more glycine may be synthesized to participate in DNA damage repair. On the other hand, with the catalysis of phosphatidylcholine (PC) synthase, serine can react with PC to generate choline and phosphatidylserine (PS). Though we did not observe PC and PS in cellular soluble metabolites upon radiation, due to their poor water solubility (no corresponding signal was observed in the hydrogen spectrum), the elevated content of choline in response to radiation indicated that the catalytic reaction occurred.

Though it has been reported that cancer cells, specially cancer stem cells, can synthesize antioxidant substances and reduce the generation of ROS to acquire radiation tolerance [39, 40], in this study, the content of the two antioxidant substances taurine and glutathione did not increase significantly in response to radiation, suggesting that radiation tolerance in B16 cells may have different mechanisms.

## Conclusions

With NMR analysis, we observed a series of change in metabolites in response to radiation in B16 cells. Based on previous studies, we concluded that these metabolites are involved in radiation tolerance in B16 cells. Taken together, our results suggested that radiation tolerance in B16 cells may result from the repair of radiation induced DNA damage.

With intensive bioinformatic analysis, NMR based metabolome analysis can be used to identify the specific metabolic pathways in response to radiation, which may provide potential targets for radiotherapy sensitization and furthermore offers technical support and theoretical evidence for personalized radiation therapy.

## Supporting Information

S1 Supporting Datasets **Dataset A.** NMR spectra using MestReNova software. **Dataset B.** Associated network generated by MetScape, a Cytoscape network file type of Fig 4. **Table A.** All pathways generated from MBRole by enrichment analysis, using metabolites in Table 2.
(ZIP)
